# Supplementary material for: Mycotoxin Decontamination Efficacy of Atmospheric Pressure Air Plasma
Source: Toxins (Basel). 2019 Apr 12;11(4):219. doi: 10.3390/toxins11040219 (PMC6521119; doi:10.3390/toxins11040219)
Supplement: Supplementary file 1 [file toxins-11-00219-s001.pdf]

Supplementary Materials: Mycotoxin Decontamination Efficacy of Atmospheric Pressure Air Plasma

Nataša Hojnik, Martina Modic, Gabrijela Tavčar-Kalcher, Janja Babič, James L. Walsh and Uroš Cvelbar

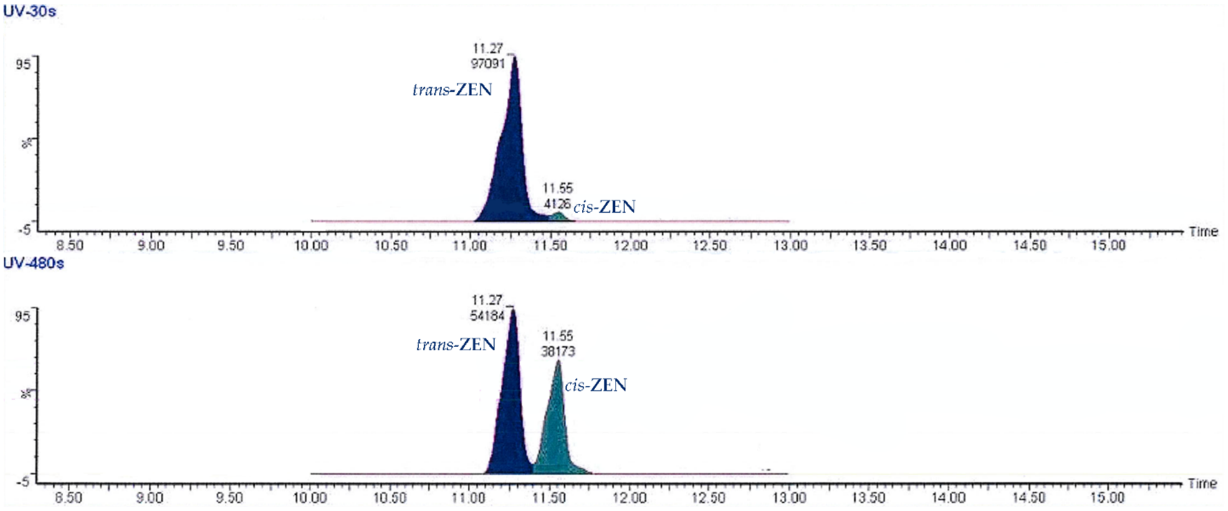

Figure 1. Chromatogram of ZEN obtained after the exposure to UVC irradiation.

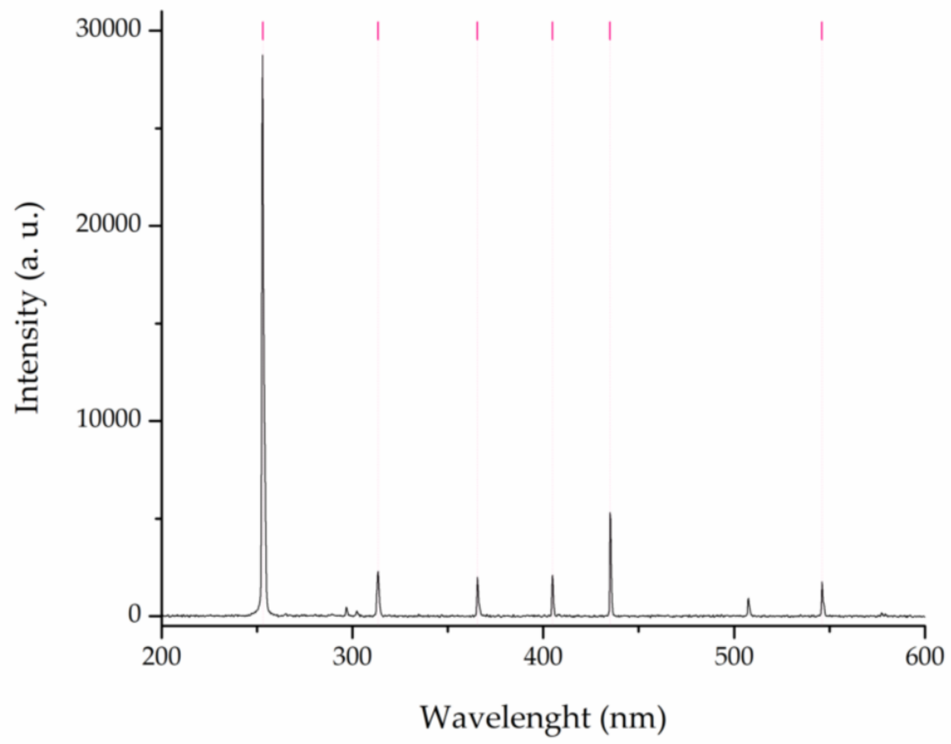

Figure 2. Hg intensity lines of UVC germicidal lamp.
